# Identification of a novel enzyme from *E. pacifica* that acts as an eicosapentaenoic 8*R*-LOX and docosahexaenoic 10*R*-LOX

**DOI:** 10.1038/s41598-020-77386-3

**Published:** 2020-11-26

**Authors:** Sayaka Yuki, Aiko Uemura, Mayuka Hakozaki, Akira Yano, Masato Abe, Yoshihisa Misawa, Naomichi Baba, Hidetoshi Yamada

**Affiliations:** 1 Iwate Biotechnology Research Center, 22-174-4 Narita, Kitakami, Iwate 024-0003 Japan; 2 Depatment of Bioscience, Graduate School of Agriculture, Ehime University, 3-5-7 Tarumi, Matsuyama, Ehime 790-8566 Japan; 3 Bizen Chemical Co., Ltd., 363 Tokutomi, Akaiwa-shi, Okayama 709-0716 Japan; 4 Faculty of Life and Environmental Science, Teikyo University of Science, 22-2-1 Senjusakuragi, Adachi-ku, Tokyo 120-0045 Japan

**Keywords:** Fatty acids, Inflammation

## Abstract

North Pacific krill (*Euphausia pacifica*) contain 8*R*-hydroxy-eicosapentaenoic acid (8*R*-HEPE), 8*R*-hydroxy-eicosatetraenoic acid (8*R*-HETE) and 10*R*-hydroxy-docosahexaenoic acid (10*R*-HDHA). These findings indicate that *E. pacifica* must possess an *R* type lipoxygenase, although no such enzyme has been identified in krill. We analyzed *E. pacifica* cDNA sequence using next generation sequencing and identified two lipoxygenase genes (*PK-LOX1* and *2*). *PK-LOX1* and *PK-LOX2* encode proteins of 691 and 686 amino acids, respectively. Recombinant PK-LOX1 was generated in Sf9 cells using a baculovirus expression system. PK-LOX1 metabolizes eicosapentaenoic acid (EPA) to 8*R*-HEPE, arachidonic acid (ARA) to 8*R*-HETE and docosahexaenoic acid (DHA) to 10*R*-HDHA. Moreover, PK-LOX1 had higher activity for EPA than ARA and DHA. In addition, PK-LOX1 also metabolizes 17*S*-HDHA to 10*R*,17*S*-dihydroxy-docosahexaenoic acid (10*R*,17*S*-DiHDHA). PK-LOX1 is a novel lipoxygenase that acts as an 8*R*-lipoxygenase for EPA and 10*R*-lipoxygenase for DHA and 17*S*-HDHA. Our findings show PK-LOX1 facilitates the enzymatic production of hydroxy fatty acids, which are of value to the healthcare sector.

## Introduction

Lipoxygenases (LOXs) are non-heme iron-containing dioxygenases that catalyze the dioxygenation of polyunsaturated fatty acids (PUFAs)^1–4^. LOXs are found in eukaryotes and can be classified based on their sequence similarity genetic and deoxygenation activity. Humans have six functional LOX genes (*ALOX15*, *ALOX15B*, *ALOX12*, *ALOX12B*, *ALOXE3*, *ALOX5*)^5^. These LOX enzymes have traditionally been classified by the position of the hydroperoxy and hydroxy fatty acids, formed from arachidonic acid (ARA)^6^. Mammalian LOXs are single polypeptide chain enzymes made up of two domains. The smaller N-terminal domain comprises several parallel and anti-parallel beta-sheets and has been implicated in the regulation of enzyme activity and membrane binding. The larger C-terminal catalytic domain consists of several helices and contains the catalytic non-heme iron localized in the putative substrate-binding pocket^7–14^. LOXs catalyze the formation of hydroxy FAs and their metabolites that act as major mediators^5,15^. Humans do not possess ALOX8, although the mouse ortholog of human ALOX15B displays arachidonic acid 8*S*-lipoxgenase activity^16^. Mouse Alox8 specifically oxidizes the eighth carbon of ARA and catalyzes the conversion of ARA to 8*S*-hydroxy-eicosatetraenoic acid (8*S*-HETE).

*Euphausia pacifica* (North Pacific krill) is the most common krill species found in the Northern Pacific Ocean and one of the few commercially harvested *Euphausiids*. We previously showed *E. pacifica* contains 8*R*-HEPE, 8*R*-HETE and 10*R*-HDHA^17^. We also showed that 8-HEPE could be produced enzymatically from EPA in *E. pacifica*^17,18^. These observations suggest that *E. pacifica* has a lipoxygenase that metabolizes EPA to 8*R*-HEPE, ARA to 8*R*-HETE and DHA to 10*R*-HDHA. The *R*-lipoxygenase is rarely found in mammal and plant species. Moreover, ALOX12B is the only *R*-lipoxygenase found in human^5^. The *R* forms of HETEs are metabolized by aspirin-acetylated cyclooxygenase or cytochrome P450 enzymes in mammals^19,20^. The genome of krill which is estimated to be about 50 Gb, has not been determined due to its huge size^21,22^. Therefore, to date, the LOX gene of *E. pacifica* had not been identified.

The DHA-derived 10,17-dihydroxy product, termed Protectin D1 (PD1), displays potent protective and anti-inflammatory actions^23–27^. Three types of 10,17-DiHDHA: 10*R*,17*S*-dihydroxy-docosa-4*Z*,7*Z*,11*E*,13*E*,15*Z*,19*Z*-hexaenoic acid, 10*R*,17*S*-dihydroxy-docosa-4*Z*,7*Z*,11*E*,13*Z*,15*E*,19*Z*-hexaenoic acid and 10*S*,17*S*-dihydroxy-docosa-4*Z*,7*Z*,11*E*,13*Z*,15*E*,19*Z*-hexaenoic acid were identified from human leukocytes and mouse exudates. 10*R*,17*S*-dihydroxy-docosa-4*Z*,7*Z*,11*E*,13*E*,15*Z*,19*Z*-hexaenoic acid is a major type in human leucocyte and defined PD1^28^. The biosynthesis of these three 10,17-DiHDHA compounds starts from DHA, which is acted upon by ALOX15 to generate PD1 by enzymatic epoxidation. 10,17*S*-dihydroxy-docosa-4*Z*,7*Z*,11*E*,13*Z*,15*E*,19*Z*-hexaenoic acid is generated by double oxidation^28^. 10*S*,17*S*-dihydroxydocosa-4*Z*,7*Z*,11*E*,13*Z*,15*E*,19*Z*-hexaenoic acid (PDX) can be produced from DHA by soybean 15-lipoxygenase^29,30^. However, until now, enzymatic production of 10*R*,17*S*-dihydroxy-docosa-4*Z*,7*Z*,11*E*,13*Z*,15*E*,19*Z*-hexaenoic acid had not been established. Several differences in bio-activity between PD1 and PDX have been previously reported^31,32^. For example, PD1 displays higher activity for blocking neutrophil infiltration than PDX^28,31^. By contrast, PDX shows higher activity for inhibiting platelet aggregation than PD1^32,33^. 10R,17S-DiHDHA produced by double dioxygenation (10R,17S-dihydroxy-docosa-4Z,7Z,11E,13Z,15E,19Z-hexaenoic acid) has equipotent activity to PD1 and PDX in terms of blocking neutrophil infiltration and inhibiting platelet aggregation, respectively^31,33^.

In this study, we identified LOX gene (*PK-LOX1* and *2*) of *E. pacifica* and qualitatively analyzed its gene product PK-LOX1.

## Results

### Identification of two lipoxygenase genes from *E. pacifica*

**Figure 1:**
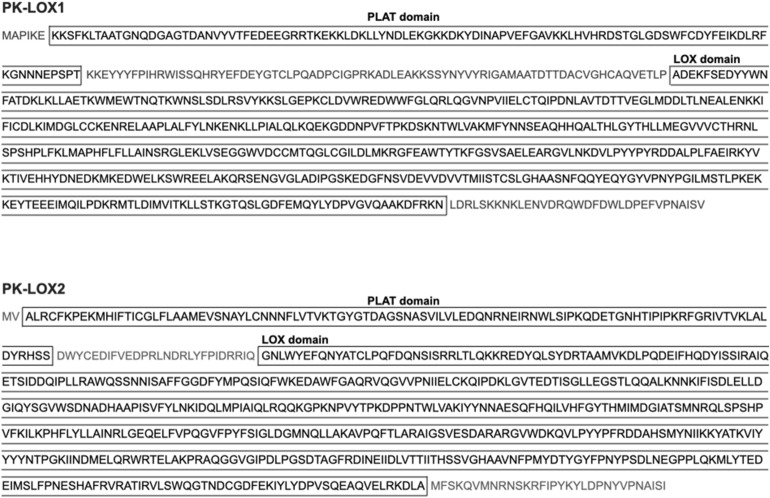
Amino acid sequences of PK-LOX1 and PK-LOX2.

We constructed 42,432 contigs from *E. pacifica* RNA sequencing reads by assemble using trinity (Supplemental data S1). Total contig size was 52,804,389. N50 contig size was 1487. A search for candidate lipoxygenase genes from 42,432 contigs using blastx identified two potential hits (*PK-LOX1* and *PK-LOX2*). *PK-LOX1* encoded a protein of 691 amino acids, which included a PLAT domain and LOX domain. *PK-LOX2* encoded a protein of 686 amino acids, which also included a PLAT domain and LOX domain (Fig. 1). The amino acid sequence homology between PK-LOX1 or PK-LOX2 and Alox8 was analyzed by blastp. The results are summarized in Supplemental Figure S1. A multiple sequence alignment of lipoxygenase proteins from North Pacific Krill, mouse, soybean and coral is shown in Supplemental Figure S6.

### Lipoxygenase activity of PK-LOX1

Recombinant baculovirus vectors were constructed for the expression of 6xHis tagged PK-LOX1, PK-LOX2, Alox8 or AcGFP. The expression level of PK-LOX1 protein was monitored every 24 h until 6 days after baculovirus infection. LOX expression peaked 48 h after infection (Supplemental Figure S2).

**Figure 2:**
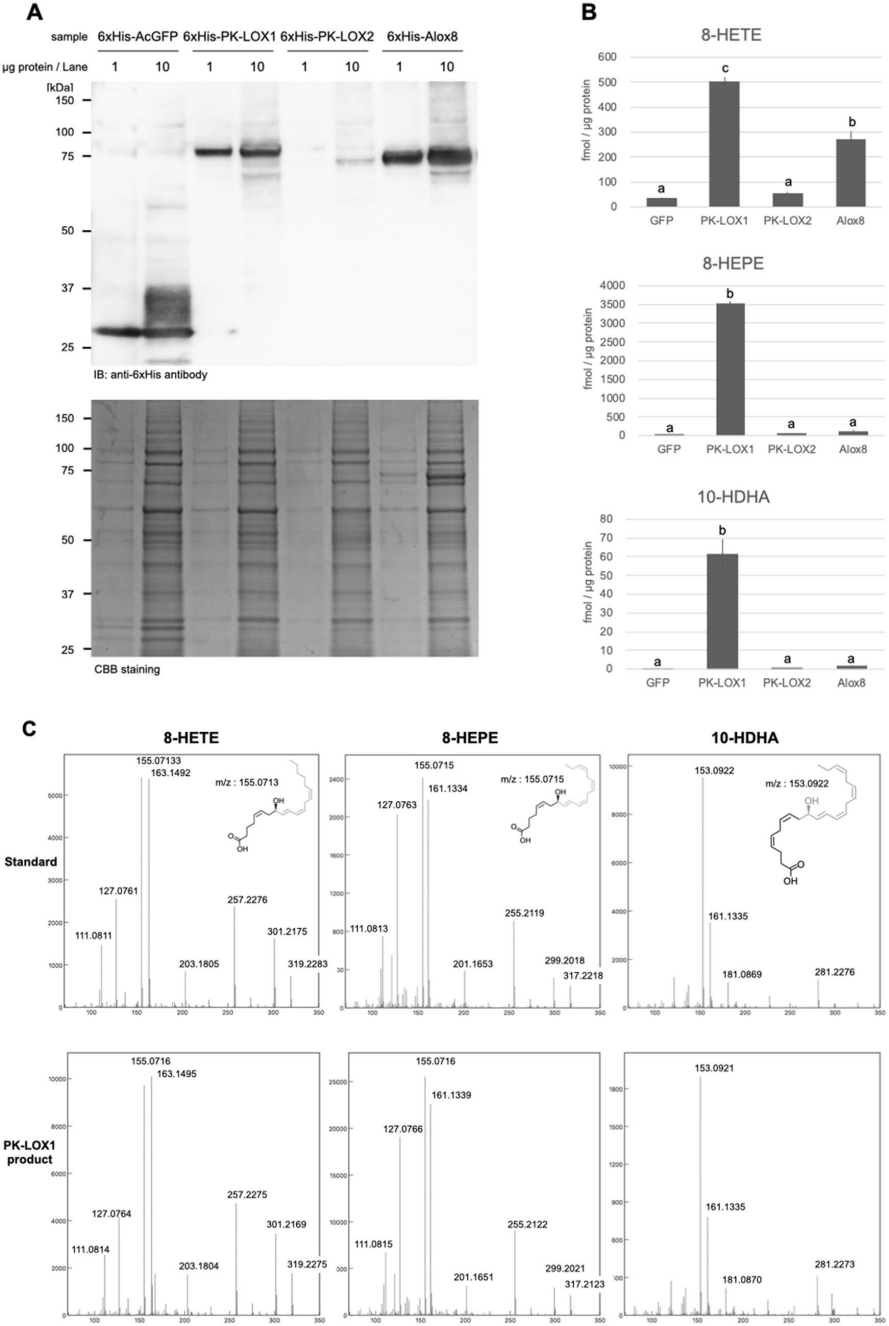
Protein expression and lipoxygenase activity of PK-LOX1 and PK-LOX2. (**A**) Western blot analysis using anti-His antibody. CBB staining was used as a loading control. (**B**) Amount of 8-HETE, 8-HEPE and 10-HDHA in solution after incubation of ARA, EPA or DHA with Sf9 cells infected with baculovirus harboring GFP, PK-LOX1, PK-LOX2 or Alox8 genes for 1 h. Values are the mean ± s.d. of three samples. Values marked by different letters were significantly different (*p* < 0.05). (**C**) MSMS spectra of 8-HETE, 8-HEPE and 10-HDHA produced by PK-LOX1.

Recombinant PK-LOX1 (80 kDa) was detected by Western blot analysis (Fig. 2A). By contrast, PK-LOX2 was barely detectable in Sf9 cells. We examined the level of RNA expression of PK-LOX1 and PK-LOX2. The expression of PK-LOX2 RNA was detected in Sf9 cells (Supplemental Table S1). Our analysis showed PK-LOX1 could produce 8-HETE, 8-HEPE and 10-HDHA from 40 µmol/L of ARA, EPA and DHA, respectively (Fig. 2B,C). Alox8 displayed the highest activity for 8-HETE production, whereas PK-LOX1 showed the highest activity for 8-HEPE production (Fig. 2B). The metabolic efficiency of 8-HETE, 8-HEPE or 10-HDHA production from 40 µmol/L of ARA, EPA or DHA by PK-LOX1 was 0.79%, 55.62% or 0.97%, respectively.

HEPE produced by PK-LOX1 from EPA was detected as a single peak by LC/QTOF analysis. By contrast, HEPE produced by Alox8 from EPA could be separated into two peaks (Fig. S3A). Specifically, Alox8 generated two product peaks corresponding to 15-HEPE and 8-HEPE (Fig. S3B). The specificity of omega 13 carbon oxidization activity of Alox8 was lower for EPA and DHA compared with ARA (Fig. S3C).

### PK-LOX1 catalyzes the stereospecific oxidation of EPA, DHA and ARA

**Figure 3:**
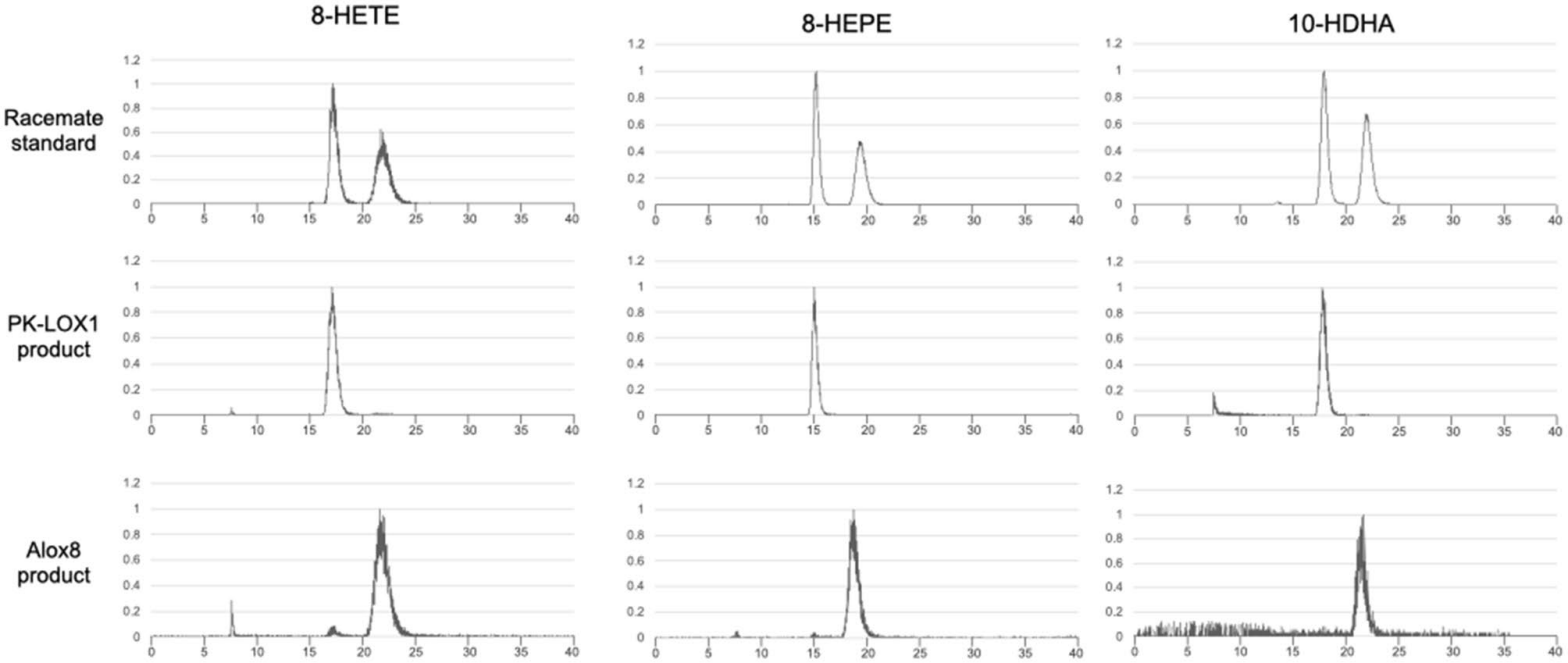
Stereochemical analysis of 8-HETE, 8-HEPE and 10-HDHA produced by PK-LOX1 or Alox8. Extracted ion chromatograms of the product ion generated from the precursor ion for 8-HETE, 8-HEPE and 10-HDHA. The product ions for 8-HETE, 8-HEPE and 10-HDHA had an *m/z* value of 155.071, 155.071 and 153.092, respectively. The corresponding precursor ions for 8-HETE, 8-HEPE and 10-HDHA had an *m/z* values of 319.2, 317.2 and 343.2, respectively.

We analyzed the stereochemical structure of 8-HETE, 8-HEPE and 10-HDHA metabolized by PK-LOX1 or Alox8 using a CHIRALPAK ID column. Previously, we demonstrated that a CHIRALPAK ID column could resolve a racemate of 8-HETE, 8-HEPE and 10-HDHA^17^. Moreover, we confirmed the second peak was the *S*-form of 8-HETE and 8-HEPE, using authentic standards of 8*S*-HETE and 8*S*-HEPE, respectively. We also concluded the second peak of rac-10-HDHA was the *S*-form, because the peak of 10-HDHA produced by Alox8 corresponded to the second peak in our previous study. Here, to establish whether the first peak of the rac-10-HDHA was 10*R*-HDHA, we reconfirmed the stereochemical structure of 10-HDHA from *E. pacifica* according to a previous report which determined the stereochemical structure of soybean LOX products using GC/MS^34^. The derivative of 10-HDHA from *E. pacifica* corresponded to the peak of (*R*)-dimethyl malate (Fig. S3). These results showed that 10-HDHA from *E. pacifica* was 10*R*-HDHA and the first peak of rac-10-HDHA was 10*R*-HDHA. The peaks of 8-HETE, 8-HEPE and 10-HDHA produced by PK-LOX1 corresponded to the first peak of the racemate standards. The peak of 8-HETE, 8-HEPE and 10-HDHA produced by Alox8 corresponded to the second peak of the racemate standards (Fig. 3). The ratio of 8R-HETE : 8S-HETE in 8-HETE produced by PK-LOX1 was 98.98 : 1.02. The ratio of 8R-HEPE : 8S-HEPE in 8-HEPE produced by PK-LOX1 was 100 : 0. The ratio of 10R-HDHA : 10S-HDHA in 10-HDHA produced by PK-LOX1 was 100 : 0. The ratio of 8R-HETE : 8S-HETE in 8-HETE produced by Alox8 was 3.51 : 96.49. The ratio of 8R-HEPE : 8S-HEPE in 8-HEPE produced by Alox8 was 1.58 : 98.42. The ratio of 10R-HDHA : 10S-HDHA in 10-HDHA produced by Alox8 was 0 : 100.

### Biochemical characterization of PK-LOX1

**Figure 4:**
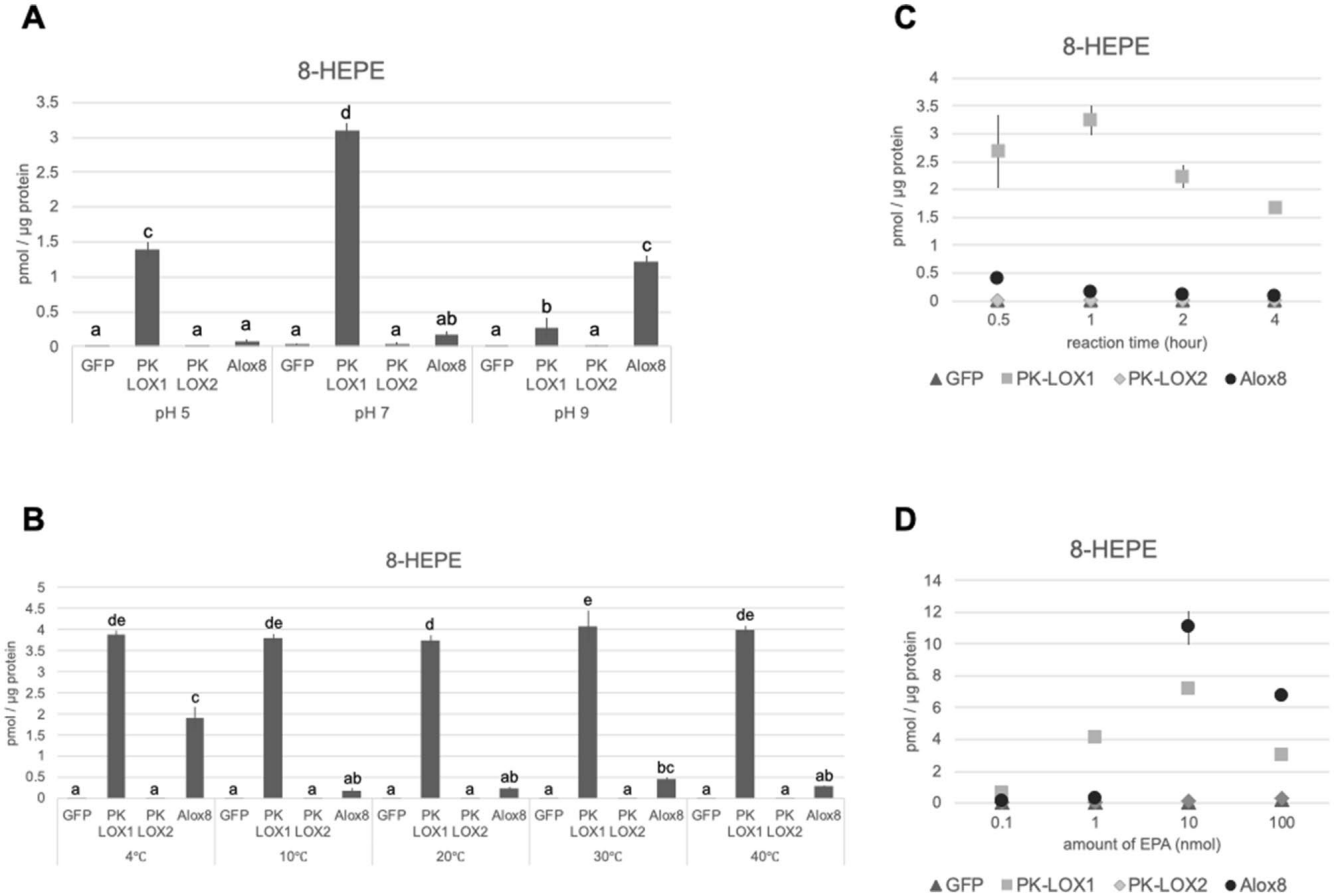
Examination of the conditions for incubation of EPA with PK-LOX1. PK-LOX1 was mixed with EPA at different pH conditions (**A**), temperatures (**B**), incubation times (**C**) and amounts of EPA (**D**). Values are the mean ± s.d. of three samples. Values marked by different letters were significantly different (*p* < 0.05).

To characterize the enzymatic properties of PK-LOX1, we examined its conversion of EPA under various conditions. PK-LOX1 displayed activity at pH 7, whereas Alox8 gave better activity under more basic conditions (Fig. 4A). In terms of temperature, PK-LOX1 gave similar levels of lipoxygenase activity at 4 to 40 °C. By contrast, Alox8 showed the highest level of activity at 4 °C (Fig. 4B). One hour was sufficient for 8-HEPE production mediated by PK-LOX1 (Fig. 4C). PK-LOX1 showed the highest enzymatic activity using 10 nmol (400 µmol/L) of EPA. Alox8 displayed more than tenfold higher activity at 10 nmol (400 µmol/L) than 1 nmol (40 µmol/L) EPA (Fig. 4D).

### PK-LOX1 can oxidize omega 13 carbon of gamma-linolenic acid (GLA), stearidonic acid (SDA) and docosapentaenoic acid (DPA)

**Figure 5:**
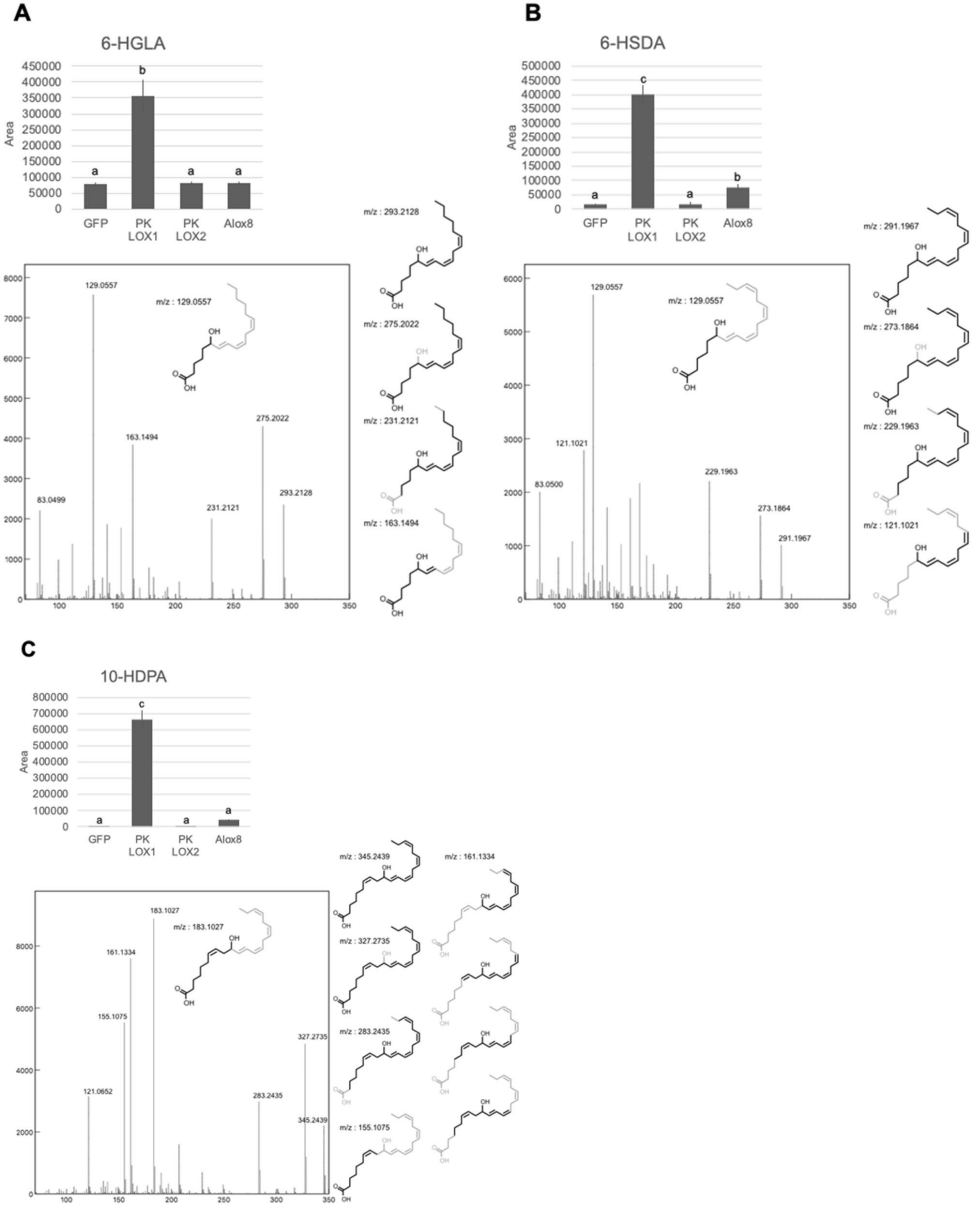
Lipoxygenase activity of PK-LOX1 for GLA, SDA and DPA. Sf9 cells infected with baculovirus harboring GFP, PK-LOX1, PK-LOX2 or Alox8 were incubated with 1 nmol of GLA (**A**), SDA (**B**) or DPA (**C**). Values of area are the mean ± s.d. of three samples. Values marked by different letters were significantly different (*p* < 0.05). MSMS spectra shown alongside the structures gave accurate mass values using SCIEX OS software.

PK-LOX1 oxidizes the omega 13 carbon of ARA, EPA and DHA. In addition GLA, SDA and DPA have a double bond at the omega 13 carbon. Here, we examined whether PK-LOX1 could oxidize these unsaturated fatty acids. The structures of hydroxy-GLA (HGLA), hydroxy-SDA(HSDA) and hydroxy-DPA (HDPA) were analyzed by LC/QTOFMS. A typical product ion of 6-HGLA (*m/z*: 129.0557), 6-HSDA (*m/z*: 129.0557) and 6-HGLA (*m/z*: 183.1027) was observed in MSMS spectra of HGLA, HSDA and HDPA produced by PK-LOX1, respectively (Fig. 5).

### Enzymatic production of 10*R*,17*S*-DiHDHA by PK-LOX1.

**Figure 6:**
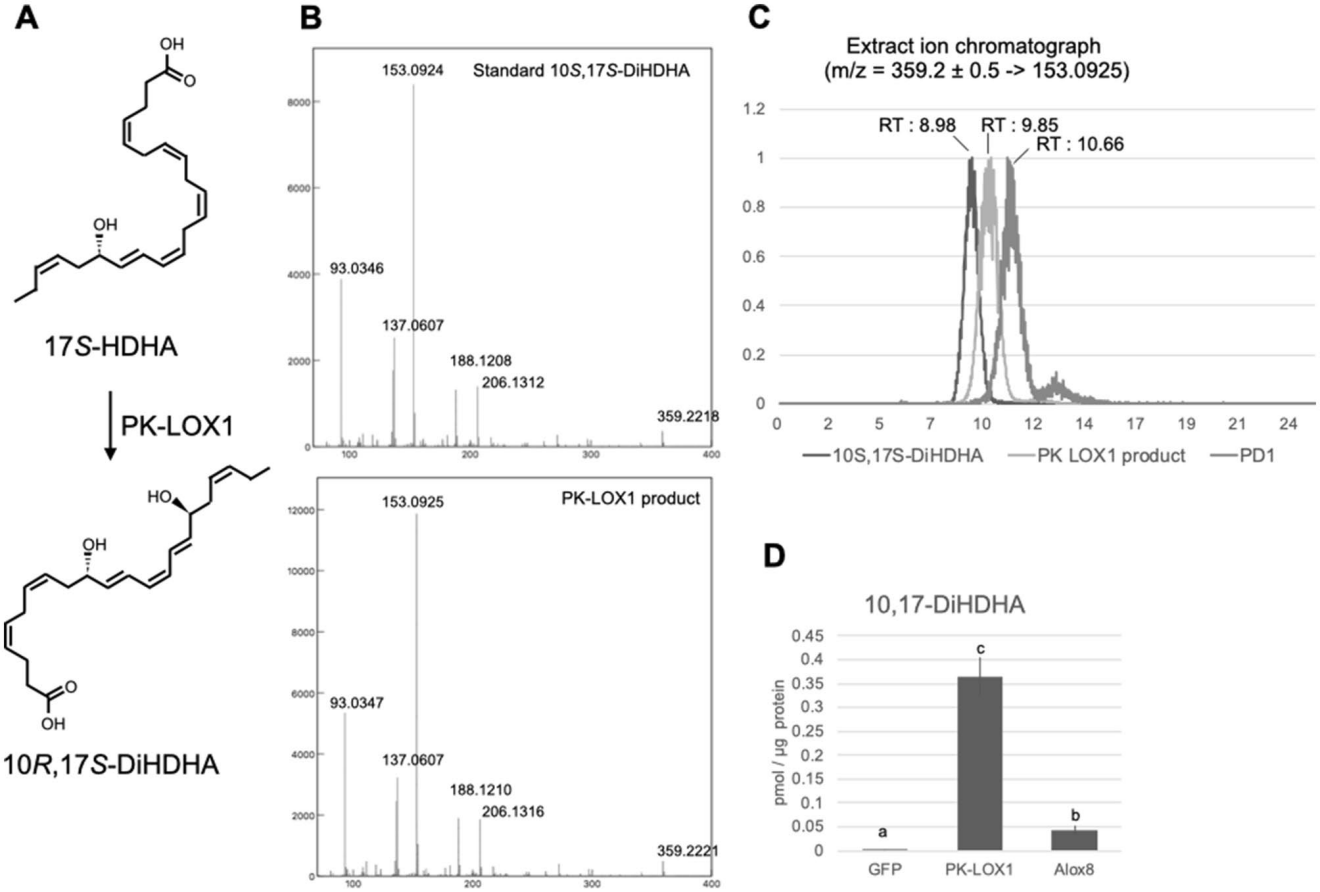
Enzymatic production of 10*R*,17*S*-DiHDHA by PK-LOX1. (**A**) Structures of 17S-HDHA and 10*R*,17*S*-DiHDHA. (**B**) MSMS spectra of 10*S*,17*S*-DiHDHA standard and DiHDHA produced by PK-LOX1 from 17*S*-HDHA. (**C**) Chromatograph of 10S,17S-DiHDA standard, 10,17*S*-DiHDHA produced by PK-LOX1 and PD1 standard. (**D**). Amount of 10,17DiHDHA in solution after incubation of 17*S*-HDHA with Sf9 cells infected with baculovirus harboring GFP, PK-LOX1 or Alox8 genes at 30 °C for 1 h. Values are the mean ± s.d. of three samples. Values marked by different letters were significantly different (*p* < 0.05).

We examined the enzymatic production of 10*R*,17*S*-DiHDHA from 17*S*-HDHA (17*S*-hydroxy-docosa-4Z,7Z,10Z,13Z,15E,19Z-hexaenoic acid) catalyzed by PK-LOX1. The structures of 17*S*-HDHA and 10*R*,17*S*-DiHDHA are shown in Fig. 6A. The DiHDHA produced by PK-LOX1 was analyzed by LC/QTOFMS using a LunaC18-2 column. The MSMS spectra of DiHDHA produced by PK-LOX1 corresponded to the MSMS spectra of 10*S*,17*S*-DiHDHA standard (Fig. 6B). We also analyzed the stereochemical structure of 10,17-DiHDHA produced from 17*S*-HDHA by PK-LOX1. The sample was compared to a previous study involving the organic synthesis of 10*R*,17*S*-DiHDHA^28^. The previous study described using a Luna C18-2 column to separate 10S,17S-dihydroxy-docosa-4Z,7Z,11E,13Z,15E,19Z-hexaenoic acid, 10R,17S-dihydroxy-docosa-4Z,7Z,11E,13Z,15E,19Z-hexaenoic acid and PD1. Here, we analyzed 10S,17S-dihydroxy-docosa-4Z,7Z,11E,13Z,15E,19Z-hexaenoic acid, PD1 and 10,17S-DiHDHA produced by PK-LOX1 on a LunaC18-2 column. These three 10,17S-DiHDHAs were resolved into three discrete peaks (Fig. 6C). We also analyzed the 11,13,15 double bound geometry of 10R,17S-DiHDHA produced by PK-LOX1 according to the method reported previously by Hansen et al.^35^ (Supplemental Fig. S5). These observations show that DiHDHA produced from 17S-HDHA by PK-LOX1 was 10*R*,17*S*-dihydroxy-docosa-4Z,7Z,11E,13Z,15E,19Z-hexaenoic acid. PK-LOX1 displayed more than fivefold higher activity to produce 10,17*S*-DiHDHA from 17*S*-HDHA than Alox8 (Fig. 6D).

## Discussion

In this study, we identified two novel lipoxygenase genes (*PK-LOX1* and *2*) from *E. pacifica* (Fig. 1). *PK-LOX1* encodes a protein of about 80 kDa (Fig. 2A) that oxidizes the omega 13 carbon of ARA, EPA, DHA, GLA, SDA and DPA (Figs. 2, 5). The lipoxygenase activity of PK-LOX1 was higher for EPA than ARA and DHA (Fig. 2B). Stereospecific oxidization of ARA, EPA and DHA mediated by PK-LOX1 generated 8*R*-HETE, 8*R*-HEPE and 10*R*-HDHA (Fig. 3). Lipoxygenase activity of PK-LOX1 was observed at neutral pH and temperatures between 4 to 40 °C (Fig. 4). PK-LOX1 also oxidizes the *R* position of the omega 13 carbon of 17*S*-HDHA to produce 10*R*,17*S*-DiHDHA (Fig. 5).

PK-LOX1 is a novel lipoxygenase that acts as an 8*R*-lipoxygenase for EPA and 10*R*-lipoxygenase for DHA. Arachidonate 8-lipoxygenase has been identified from mouse (*Mus musculus*)^16^ and coral (*Plexaura homomalla*)^36^, however eicosapentaenoic 8-lipoxygenase and docosahexaenoic 10-lipoxygenase had not been identified. We compared the main differences between PK-LOX1 and Alox8. Alox8 displayed only about 12% of the carbon specific oxidation activity for ARA compared with PK-LOX1. However, Alox8 acted as an 8/15-lipoxygenase for EPA and 10/17-lipoxygenase for DHA (Fig. S3). PK-LOX1 displayed an eighth and a tenth of the carbon specific oxidation activity for EPA and DHA, respectively. Moreover, Alox8 is an *S* type lipoxygenase, whereas PK-LOX1 is an *R* type lipoxygenase (Fig. 3). *S* type lipoxygenases are common in mammals and plants, but *R* type lipoxygenases tend to be confined to marine organisms. For example, arachidonate 8*R*-lipoxygenase was identified from coral^36^, and 11*R* and 12*R*-lipoxygenase activity were found in the eggs of sea urchins^37^. Here, we identified 8*R*-lipoxygenase from North Pacific krill, which is consistent with the suggestion that *R* type lipoxygenases are more common in marine organisms.

In this study, we show that PK-LOX1 can generate 10*R*,17*S*-DiHDHA from 17*S*-HDHA (Fig. 6). The enzymatic production of 17*S*-HDHA and 10*S*,17*S*-DiHDHA has previously been demonstrated using soybean lipoxygenase^29,30^. However, this study is the first to report the enzymatic production of 10*R*,17*S*-DiHDHA. Previous work showed 10,17-DiHDHA displays an anti-inflammatory effect^23–27^, but the structure–activity relationship of this compound was not determined. The method outlined in this study for the enzymatic production of 10*R*,17*S*-DiHDHA using PK-LOX1 will facilitate investigations into the structure–activity relationship.

Although krill is a key species in marine ecosystems, there is a paucity of genome data for these organisms. Currently, the only available gene database of krill is the transcriptome analysis of Antarctic krill (*Euphausia superba*)^22^. Thus, the contig data of *E. pacifica* constructed in this study is a new source of gene information for krill. Nonetheless, the biological function of 8-HEPE in *E. pacifica* remains unclear. The lipoxygenase gene sequence from *E. pacifica* will be useful in studying the physiological role of 8-HEPE in krill.

In summary, we have identified two novel lipoxygenases from *E. pacifica*. One of these lipoxygenases mediates the enzymatic production of hydroxy fatty acids that are beneficial for human health. We believe the identification and characterization of lipoxygenases from various species will form the basis of a novel method for the stereochemical specific synthesis of oxidized fatty acids. As such, marine organisms are a potential source of novel types of lipoxygenase activity.

## Material and methods

### Materials

ARA, EPA, and DHA were purchased from Nacalai Tesque (Kyoto, Japan). (±)8-HEPE (comprising equal amounts of 8*S*-HEPE and 8*R*-HEPE), 8*S*-HEPE, (±)8-HETE, 8*S*-HETE, 8*R*-HETE, rac-10-HDHA, PD1, gamma-linoleic acid (GLA), stearidonic acid (SDA) and docosapentaenoic acid (DPA) were purchased from Cayman Chemical Company (Ann Arbor, MI, USA). The *Spodoptera frugiperda* (Sf9) insect cells, and pFastBac vector were purchased from Thermo Fisher Scientific (Franklin, MA, USA). The anti-6xHis antibody [HIS.H8] was purchased from Abcam Japan (Tokyo, Japan).

### Determination of de novo RNA sequence

Total RNA was purified from *E. pacifica* using an RNeasy Lipid tissue mini kit (Qiagen, Tokyo, Japan). The library of *E. pacifica* for next generation sequencing was made using a TruSeq RNA library prep kit v2 (Illumina, Tokyo, Japan). RNA purification and library preparation were performed according to the manufacturers’ instructions. The library was analyzed by Miseq using a Miseq reagent kit v3 (600 cycle) (Illumina). The fastaq data was assembled by Trinity^38^. Contigs encoding similar amino acid sequences to human and mouse lipoxygenases were identified using blastx^39^. Our analysis identified two such contigs, which were named *PK-LOX1* and *PK-LOX2*.

### *PK-LOX1* and *PK-LOX2* cloning

Double strand cDNA was synthesized using a PrimeScript Double Strand cDNA Synthesis Kit (Takara, Shiga, Japan). *PK-LOX1* was amplified by PCR using KOD plus neo (TOYOBO, Osaka, Japan) and gene specific primers (*PK-LOX1*: 5′-GGAATCAAATCATAATGGCGCCA-3′ and 5′-AGCTTGTTTTATACACTGATGGCA-3′, *PK-LOX2*: 5′-TTGGTAACGCTGGCTCAGTC-3′ and 5′-ACTGACTAAATACTTATTGCATTTGGA-3′). The PCR products were cloned into pUC19 vector using a TOPO blunt cloning kit. The *PK-LOX1* and *PK-LOX2* cDNA sequences were confirmed by Sanger sequencing.

### Construction of baculovirus

*PK-LOX1* cDNA on pUC19 vector was amplified using the forward primer (5′-TGTATTTTCAGGGCGCCATGGCGCCAATTAAGGAAAAGAA-3′) that had homologous DNA sequence to ligate to 6xHis-TEV DNA and the reverse primer (5′-AGTGAGCTCGTCGACGTAGGCTATACACTGATGGCATTTGGAA-3′) that had homologous DNA sequence to ligate to pFastBac vector. *PK-LOX2* cDNA on pUC19 vector was amplified using the forward primer (5′-TGTATTTTCAGGGCGCCATGGTAGCGCTGCGCTGCTTCAA-3′) that had homologous DNA sequence to ligate to 6xHis-TEV DNA and the reverse primer (5′-AGTGAGCTCGTCGACGTAGGCTAAATACTTATTGCATTTGGAA-3′) that had homologous DNA sequence to ligate to pFastBac vector. The *PK-LOX* DNA sequences were cloned into pFastBac1 vector at the Stu1 restriction enzyme site together with the DNA encoded 6xHis-TEV (ATGTCGTACTACCATCACCATCACCATCACGATTACGATATCCCAACGACCGAAAACCTGTATTTTCAGGGCGCCATG) using NEBuilder (NEB, Ipswich, MA, USA). We constructed bacmid including 6xHis-TEV-PK-LOX1 or 6xHis-TEV-PK-LOX2 using the Bac-to-Bac Baculovirus Expression System (Thermo Fisher Scientific).

### Cell culture and baculovirus infection

Sf9 cells were cultured with Grace's medium (Thermo Fisher Scientific) containing 10% heat-inactivated fetal bovine serum (Thermo Fisher Scientific) and antibiotic–antimycotic solution at 27 °C. We transfected 1 µg of Bacmid DNA into 8 × 10^5^ Sf9 cells using 6 µL of Cellfectin II Reagent (Thermo Fisher Scientific). After 7 days culture, media was collected as the first passage (P1) of recombinant baculovirus stock. The titer of P1 virus stock was measured using BacPAK qPCR Titration Kit (Takara). The baculovirus stock was amplified to infect Sf9 cells with P1 virus (MOI = 1). We infected baculovirus including 6xHis-AcGFP, 6xHis-PK-LOX1, 6xHis-PK-LOX2 or 6xHis-Alox8 genes into Sf9 cells. After 2 days of culture, cells were collected and stored at − 80 °C to use for analysis of lipoxygenase activity.

### Western blot analysis

The details of Western Blot analysis was described previously^40^. Briefly, total protein was extracted from baculovirus infected Sf9 cells using RIPA lysis buffer containing protease and phosphatase inhibitor cocktail (Thermo Fisher Scientific). The protein concentration of supernatant was determined by Bradford assay. One µg of protein was subjected to electrophoresis using a NuPAGE 4–12% Bis–Tris Protein gel (Thermo Fisher Scientific). Anti-6xHis antibody was used for His-tagged protein detection.

### Enzyme reaction of lipoxygenase

For enzymatic analysis of lipoxygenase (Figs. 2, 3, 4), we infected baculovirus with 2.5 × 10^7 of Sf9 cells (MOI = 1) and cultured them for 2 days. Cells were collected, divided into 60 microtubes and stored at − 80 °C until required. The stored cell pellet was re-ssuspended in 25 µL of 200 mM Tris–HCl buffer containing 1 nmol of EPA, ARA or DHA and incubated under a variety of conditions (see later). After incubation, 75 µL of acetonitrile containing 1% (v/v) formic acid were added, vortexed and centrifuged at 20,000*g* for 10 min at room temperature. The supernatant was analyzed by Liquid chromatography/hybrid quadrupole time-of-flight mass spectrometry (LC/QTOFMS). The total amount (mol) of 8-HETE, 8-HEPE and 10-HDHA in the supernatant was determined and then normalized according to the total protein (µg) in the microtube. Total protein in the microtube that contained GFP, PK-LOX1, PK-LOX2 or Alox8 infected Sf9 cells was 223.2, 158.6, 126.9 or 161.5 µg, respectively. The metabolic efficiencies of ARA to 8-HETE, EPA to 8-HEPE and DHA to 10-HDHA were calculated by the amount of 8-HETE, 8-HEPE or 10-HDHA in the supernatant (nmol)/the additional amount of ARA, EPA or DHA (1 nmol).

### Analysis of the lipoxygenase products

We used LC/QTOFMS (Triple TOF 5600, SCIEX) for qualitative and quantitative analyses of the lipoxygenase products. The conditions used for LC/QTOFMS were described previously^17^. We used the multiple reaction monitoring (MRM) method to quantify 8-HEPE (m/z of precursor ion: 317.2 and product ion: 155.071), 8-HETE (m/z of precursor ion: 319.2 and product ion: 155.071), 10-HDHA (m/z of precursor ion: 343.2 and product ion: 153.092) and 10,17S-DiHDHA (m/z of precursor ion: 359.2 and product ion: 153.0925).

### LC/QTOFMS analysis using a chiral column

We previously described detailed methods of stereochemical analysis of 8-HEPE, 8-HETE and 10-HDHA^17^. Briefly, the enantiomers of 8-HETE, 8-HEPE and 10-HDHA were separated on a CHIRALPAK ID (4.6 mm dia. × 250 mm column; DAICEL, Osaka, Japan) with isocratic elution (formic acid solution pH 2.0/methanol, 20/80).

### Stereochemical analyses of 10*S*, 17*S*-DiHDHA and 10*R*, 17*S*-DiHDHA

The previous study showed that a Luna C18-2 column could be used to separate 10*R*,17*S*-dihydroxy-docosa-4*Z*,7*Z*,11*E*,13*Z*,15*E*,19*Z*-hexaenoic acid and 10*S*,17*S*-dihydroxy-docosa-4Z,7Z,11E,13Z,15E,19Z-hexaenoic acid. We used a Luna C18-2 column (4.6 mm dia. × 150 mm column) with gradient elution (water containing 0.1%(v/v) formic acid/acetonitrile containing 0.1%(v/v) formic acid, 35/65 hold at 1 min, 35/65 to 30/70 in 10 min, 30/70 to 15/85 in 10 min, 15/85 to 0/100 4 min) at a flow rate of 0.3 mL/min. The temperature of the column was maintained at 40 °C. The compounds were identified by QTOFMS using SCIEX OS Software. We used the same MS conditions as the quantification method for chiral analysis.

### Statistical analysis

Statistically significant differences between the experimental groups were identified using one-way ANOVA and Tukey’s post-hoc tests.

## Supplementary information

Supplementary Information.

## Data Availability

The relevant data are available at Mendeley Data (https://data.mendeley.com). The dataset of de novo RNA sequencing is available at https://dx.doi.org/10.17632/f2xhbjhddj.2. The other data is available at https://dx.doi.org/10.17632/m7hptyjnvb.1.

## Abbreviations

LOX: Lipoxygenase
EPA: Eicosapentaenoic acid
DHA: Docosahexaenoic acid
ARA: Arachidonic acid
HEPE: Hydroxy-eicosapentaenoic acid
HDHA: Hydroxy-docosahexaenoic acid
HETE: Hydroxy-eicosatetraenoic acid
MeOH: Methanol
PUFA: Polyunsaturated fatty acid
LC/QTOFMS: Liquid chromatography/hybrid quadrupole time-of-flight mass spectrometry
