# Supplementary material for: Identification of a novel enzyme from E. pacifica that acts as an eicosapentaenoic 8R-LOX and docosahexaenoic 10R-LOX
Source: Sci Rep. 2020 Nov 26;10:20592. doi: 10.1038/s41598-020-77386-3 (PMC7693274; doi:10.1038/s41598-020-77386-3)
Supplement: Supplementary file 1 — Supplementary Information. [file 41598_2020_77386_MOESM1_ESM.docx]

**Title**

Identification of a novel enzyme from *E. pacifica* that acts as an eicosapentaenoic 8*R*- LOX and docosahexaenoic 10*R*-LOX

**Authors**

Sayaka Yuki^1†^, Aiko Uemura^1^, Mayuka Hakozaki^1^, Akira Yano^1^, Masato Abe^2^, Yoshihisa Misawa^3^, Naomichi Baba^3^ and Hidetoshi Yamada^1,4†^*.

**Affiliations**

^1^Iwate Biotechnology Research Center, 22-174-4 Narita, Kitakami, Iwate 024-0003, Japan

^2^Depatment of Bioscience, Graduate School of Agriculture, Ehime University, 3-5-7 Tarumi, Matsuyama, Ehime 790-8566, Japan

^3^Bizen Chemical Co., Ltd., 363 Tokutomi, Akaiwa-shi, Okayama 709-0716, Japan

^4^Faculty of Life & Environmental Science, Teikyo University of Science, 22-2-1 Senjusakuragi, Adachi-ku, Tokyo 120-0045, Japan

**Equal contributions**

^†^These authors contributed equally.

**Correspondence**

Dr. Hidetoshi Yamada, Faculty of Life & Environmental Science, Teikyo University of Science, 22-2-1 Senjusakuragi, Adachi-ku, Tokyo 120-0045, Japan Tel.: +81 3 6910 3741 E-mail address: hyamada@ntu.ac.jp

Supplemental Figure S1


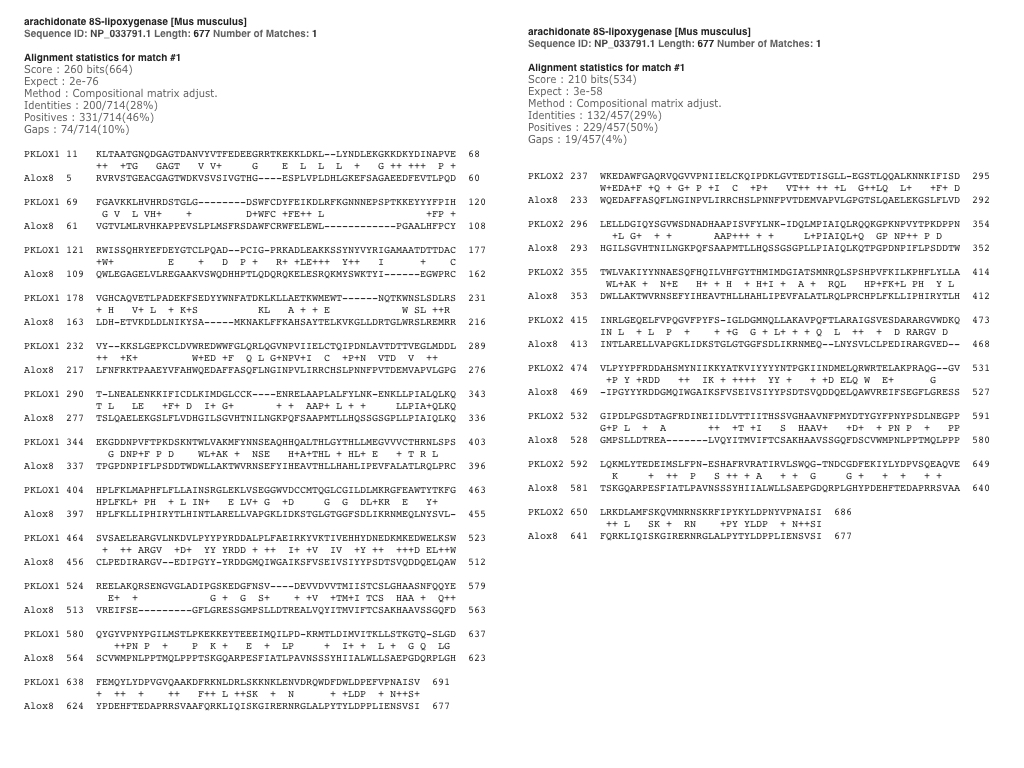


Supplemental Figure S1. Homology of amino acid sequences between Alox8 and either PK-LOX1 or PK-LOX2.

Supplemental Figure S2


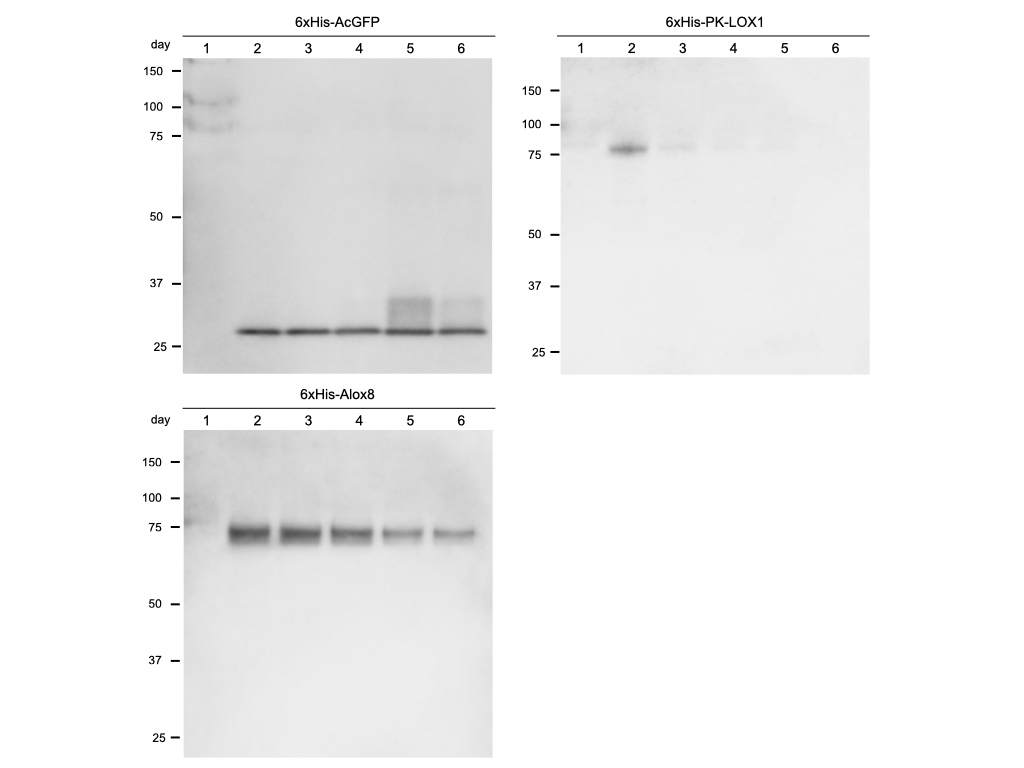


Supplemental Figure S2. Protein expression of His tagged AcGFP, PK-LOX1 and Alox8.

One µg of protein extracted from baculovirus infected Sf9 cells was analyzed by sodium dodecyl sulfate polyacrylamide gel electrophoresis. His tagged proteins were detected using an anti-6xHis antibody.

Supplemental Figure S3.


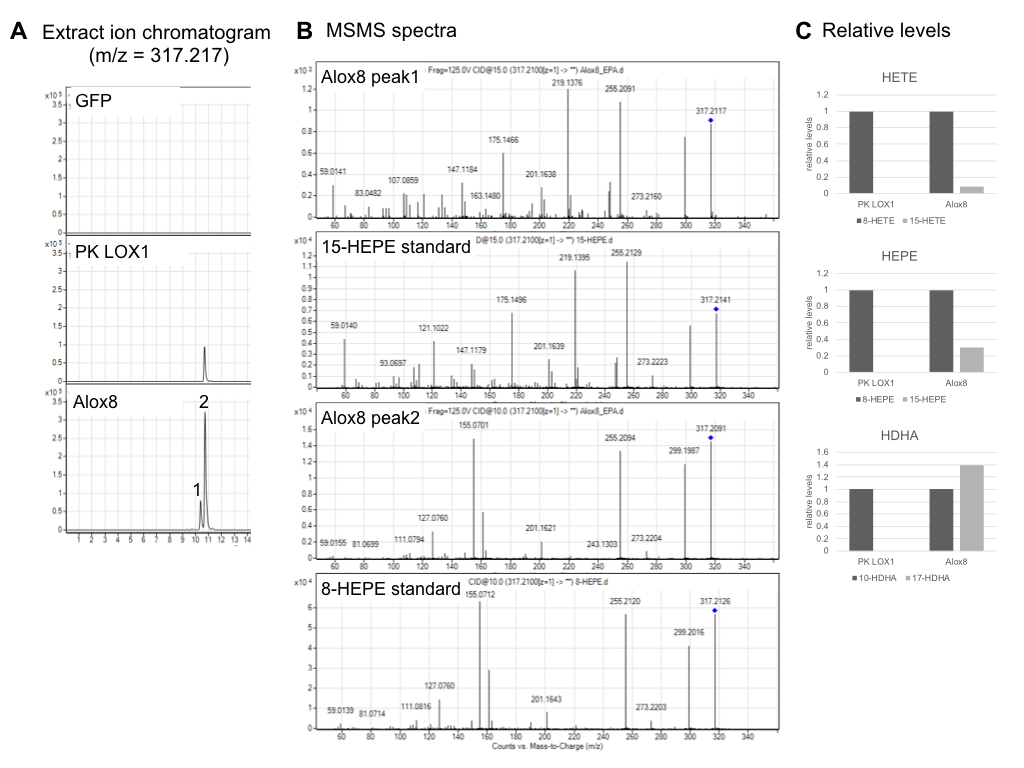


Supplemental Figure S3. Extracted ion chromatogram and MS/MS spectra of HEPEs metabolized by Alox8 from EPA. (A) Extracted ion chromatogram of HEPE (m/z = 317.217). (B) MS/MS spectra of HEPEs metabolized by Alox8. (C) Relative level of 15-HETE, 15-HEPE and 17-HDHA to 8-HETE, 8-HEPE and 10-HDHA, respectively.

Supplemental Figure S4


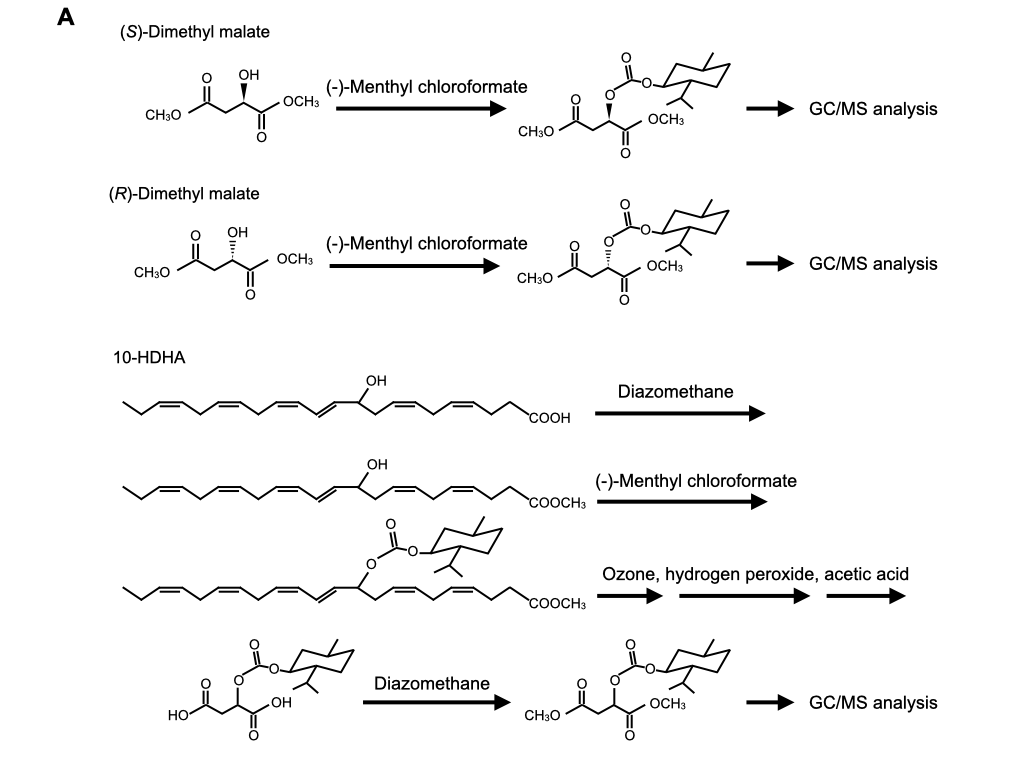


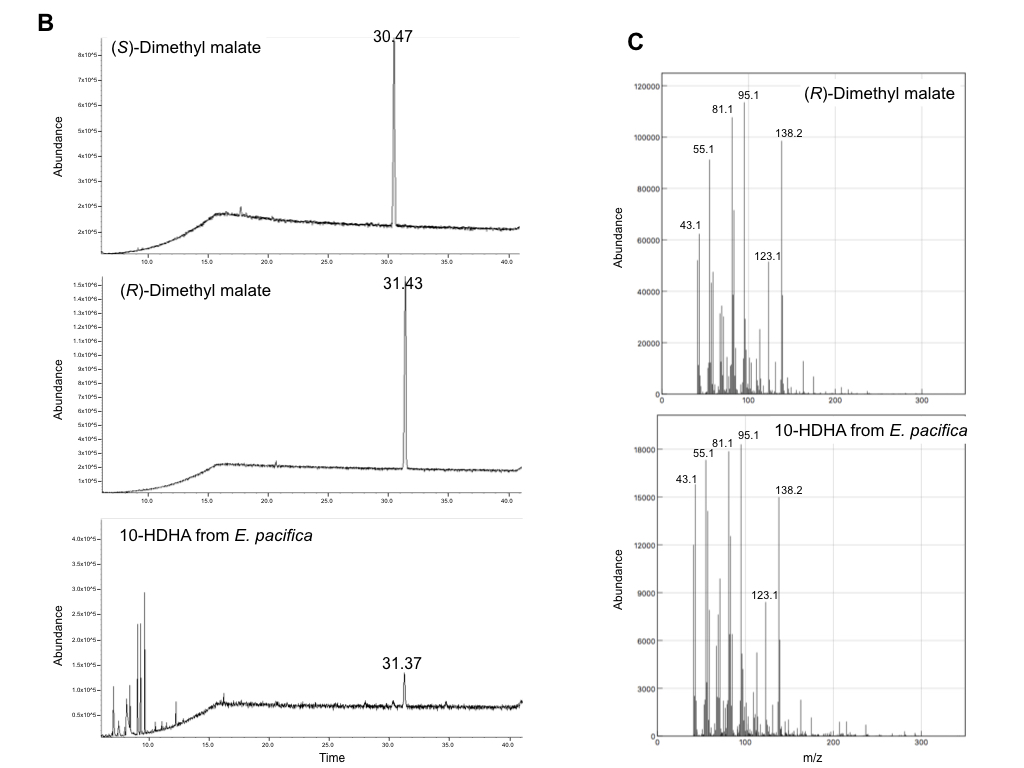


Supplemental Figure S4. Stereochemical analysis of 10-HDHA from *E. pacifica*.

(A) Scheme of derivatization. (B) Total ion chromatograph of GC/MS. (C) Mass spectra of derivatives of *(R*)-dimethyl malate and 10-HDHA from *E. pacifica*.

Supplemental Figure S5


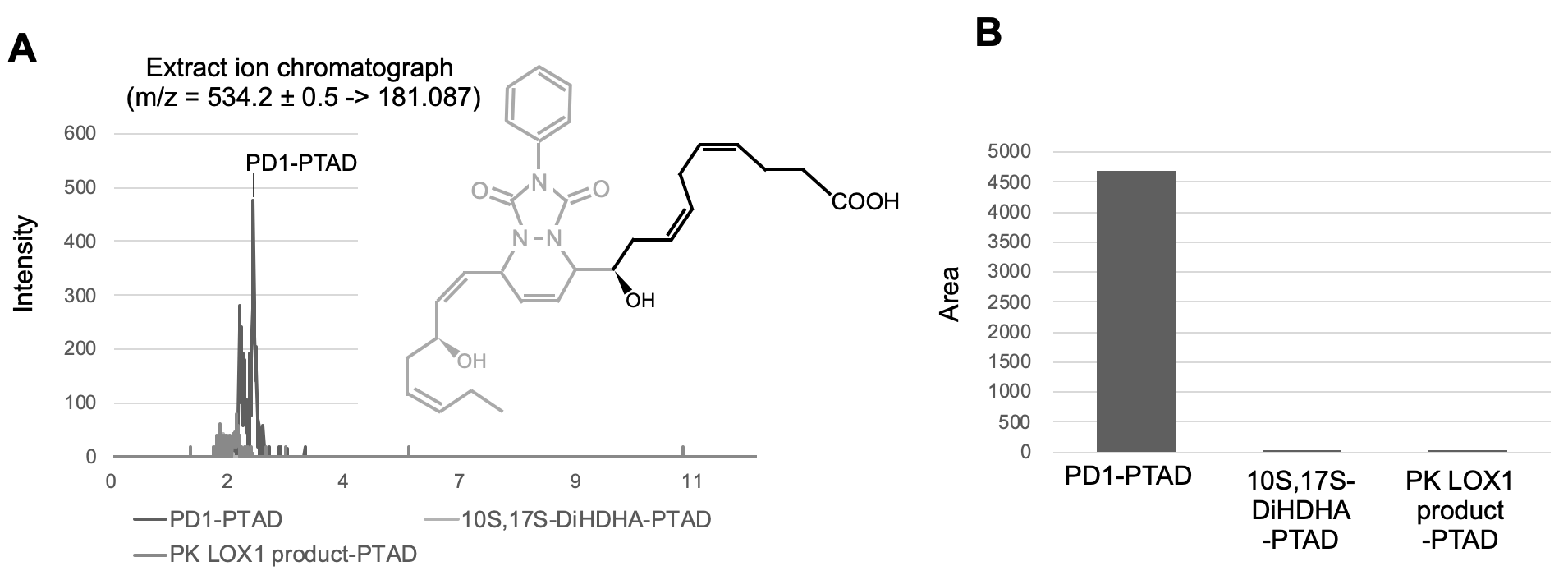


Supplemental Figure S5. Analysis of 11,13,15 double bound geometry

10*R*,17*S*-DiHDHA produced by PK-LOX1 was purified from the supernatant of the PK-LOX1 and 17*S*-HDHA reaction mixture by preparative HPLC. 10R,17S-DiHDHA was separated on an InertSustain ODS-3 column (20 mm dia. × 250 mm, GL Science Inc.) by gradient elution (water containing 0.1% formic acid/acetonitrile containing 0.1% formic acid, 55/45 to 0/100 in 20 min) at a flow rate of 15 mL/min. Compounds in the eluate were detected at 275 nm. A 15 µL aliquot of 83 mg/mL 4-phenyl-1,2,4-triazole-3,5-dione (PTAD purchased from Sigma-Aldrich Japan, Tokyo, Japan) in dichloromethane was mixed with 285 µL of 1 µmol/L PD1, 10*S*,17*S*-DiHDHA or PK-LOX1 product in ethanol. The reaction mixtures were incubated on ice for 30 minutes, diluted 10-fold in ethanol (v/v) and then subjected to LC/QTOFMS analysis.


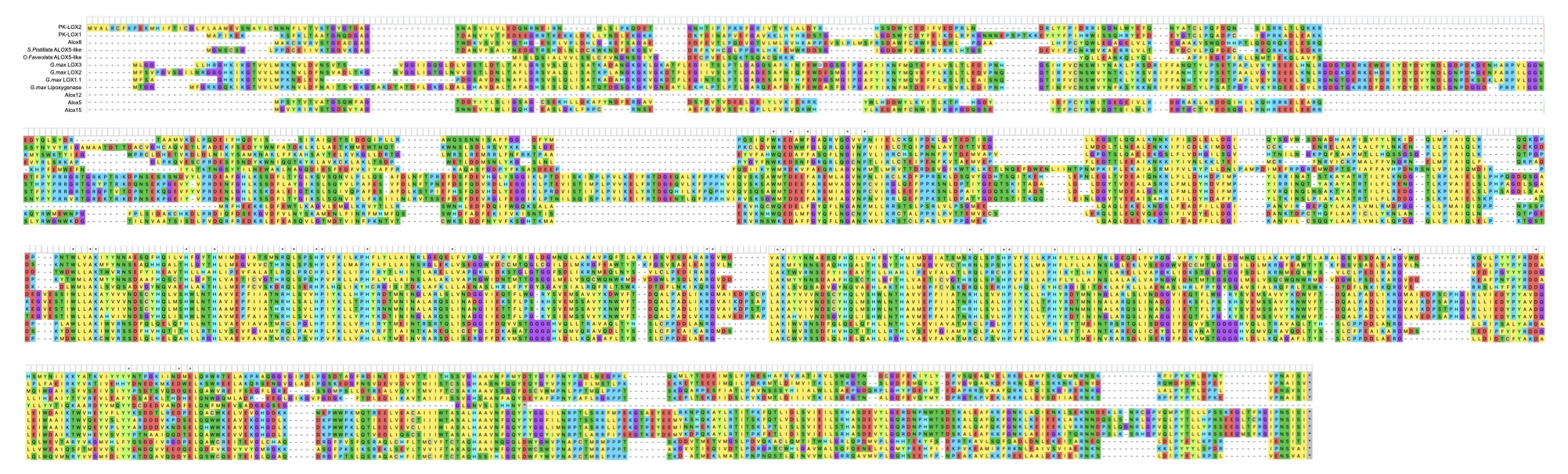


Supplemental Figure S6. A multiple sequence alignment of LOX proteins

The amino acids sequences of PK-LOX1, PK-LOX2, Alox8 (NM_009661.4), Alox5 (NM_009662.2), Alox12 (NM_001331118.1), Alox15 (NM_009660.3), *G.max* Lipoxygenase (NM_001248260.1), *G.max* LOX1.1 (NM_001249224.2), *G.max* LOX2 (NM_001250756.2), *G.max* LOX3 (NM_001248454.2), *Stylophora pistillata* arachidonate 5-lipoxygenase-like (XM_022948230.1) and *Orbicella faveolata* arachidonate 5-lipoxygenase-like (XM_020768965.1) were aligned by MEGAX software (<https://www.megasoftware.net>).

Supplemental Table 1

The RNA expression of PK-LOX1 and PK-LOX2 in Sf9 cells

| Infected baculovirus | Days after infection | Ct value of real-time PCR | |
| --- | --- | --- | --- |
|  |  | PK-LOX1 | PK-LOX2 |
| 6xHis-GFP | Day 1 | 37.0 ± 0.6 | 36.1 ± 5.4 |
|  | Day 2 | 36.5 ± 0.6 | 30.2 ± 1.4 |
|  | Day 3 | 34.5 ± 1.0 | 34.5 ± 2.1 |
|  | Day 4 | 33.6 ± 0.5 | 28.2 ± 0.2 |
| 6xHis-PK-LOX1 | Day 1 | 17.0 ± 0.7 | 37.6 ± 3.2 |
|  | Day 2 | 13.5 ± 0.4 | 32.4 ± 1.6 |
|  | Day 3 | 13.9 ± 0.1 | 29.4 ± 0.9 |
|  | Day 4 | 13.7 ± 0.1 | 28.7 ± 0.2 |
| 6xHis-PK-LOX2 | Day 1 | 36.0 ± 0.1 | 12.8 ± 0.2 |
|  | Day 2 | 32.7 ± 1.5 | 9.9 ± 0.4 |
|  | Day 3 | 32.9 ± 2.0 | 10.6 ± 0.3 |
|  | Day 4 | 32.3 ± 1.9 | 10.6 ± 0.3 |
| 6xHis-Alox8 | Day 1 | 37.5 ± 0.9 | 31.2 ± 1.1 |
|  | Day 2 | 33.7 ± 2.4 | 30.0 ± 0.6 |
|  | Day 3 | 33.3 ± 0.8 | 27.4 ± 1.7 |
|  | Day 4 | 35.2 ± 0.8 | 29.3 ± 0.8 |

Total RNAs were extracted with an RNeasy kit (QIAGEN, Tokyo, Japan) and used to synthesize cDNAs using a PrimeScript RT reagent kit (Takara, Shiga, Japan); all kits were used according to the manufacturers’ recommendations. Quantitative real-time PCR was performed with the gene specific primers (PK-LOX1 forward primer: 5’- TATGGCACCGCACTTCTTGT-3’, PK-LOX1 reverse primer: 5’- ACCCATCCACCTTCGGAAAC-3’, PK-LOX2 forward primer: 5’- TTTGTGCCCCAAGGAGTGTT-3’, and PK-LOX2 reverse primer: 5’- CCCTGGCGTCAGATTCAACT-3’) and Fast SYBR Green master mix (Applied Biosystems, Foster City, CA, USA). All quantitative real-time PCR experiments were performed using 4 ng of cDNA.
